# Comparison of vascularization and overall perfusion of the bladder wall between women with and without overactive bladder syndrome

**DOI:** 10.1038/s41598-020-64532-0

**Published:** 2020-05-05

**Authors:** Sheng-Mou Hsiao, Jin-Chung Shih, Chien-Nan Lee, Ho-Hsiung Lin

**Affiliations:** 1 Department of Obstetrics and Gynecology, Far Eastern Memorial Hospital, Banqiao, New Taipei Taiwan; 2 Department of Obstetrics and Gynecology, National Taiwan University College of Medicine and Hospital, Taipei, Taiwan; 3 Graduate School of Biotechnology and Bioengineering, Yuan Ze University, Taoyuan, Taiwan

**Keywords:** Bladder, Urological manifestations

## Abstract

The pathophysiology of female overactive bleeder syndrome (OAB) remains undetermined. Our aim is to elucidate the role of vacularization and overall perfusion of the bladder wall in women with OAB. Between 2010 and 2016, women with OAB and the asymptomatic controls were enrolled. Women with OAB were treated with tolterodine. Women with OAB (n = 40) had higher vascularization index (0.40 ± 0.57 versus 0.17 ± 0.22, p = 0.003), vascularization-flow index (0.15 ± 0.28 versus 0.05 ± 0.08, p = 0.003) and thicker trigone (0.56 ± 0.13 cm versus 0.47 ± 0.11 cm, p = 0.004), compared with the controls (n = 34). The following optimum cut-off values to predict OAB were determined: (1) vascularization index (%) ≥ 0.16, (2) vascularization-flow index ≥ 0.032, and (3) trigone bladder wall thickness ≥ 0.47 cm with an area under the curve of 0.71, 0.71 and 0.70, respectively. Correlation analysis showed that a significant correlation between urgency and vascularization index/vascularization-flow index (Spearman’s rho = 0.34 and 0.35, respectively, all p < 0.01). However, after 12 weeks of tolterodine treatment, the vascularization index, flow index and vascularization-flow index did not differ between baseline and after treatment. In conclusion, women with OAB have higher vascularization and overall perfusion of the bladder wall, compared women without OAB. However, vascularization and overall perfusion did not change after antimuscarinic treatment.

## Introduction

Overactive bladder syndrome (OAB) is defined with the presence of urinary urgency, and the absence of urinary tract infection or other obvious pathology^1^. OAB is usually accompanied by frequency and nocturia^1^.

Ischemia is considered a possible etiology of female OAB^2^. Chronic ischemia was reported to be associated with severity of lower urinary tract symptoms^3,5–7^. Ponholzer *et al*. reported that the total score of International Prostate Symptom Score (IPSS) increased with more vascular risk factors in women^3^. IPSS storage subscore was reported to be positively correlated with severity of female OAB symptoms^4^. Thus, vascular risk factors seem to be associated with female OAB. Pinggera *et al*. also reported that daytime frequency and nocturia were negatively correlated with blood perfusion in the urinary bladder^5^. Besides, a mouse model of nonneurogenic detrusor overactivity caused by systemic atherosclerosis has been successfully established^6^. Nonetheless, there is a paucity of studies, which compare the vascularization and blood perfusion of the bladder wall between women with and without OAB.

Bladder wall thickness (BWT) and detrusor wall thickness have been used to assess women with OAB^8–16^. Nonetheless, Kojima *et al*. used ultrasound-estimated bladder weight as a measure of bladder hypertrophy^17^. By multiplying the bladder wall volume (BWV) by the specific gravity of the bladder wall tissue, one can obtain the ultrasound-estimated bladder weight^17^. In our study, we estimated BWV with a novel method, instead of ultrasound-estimated bladder weight.

Women with OAB frequency have sexual dysfunction^18^. However, Ergenoglu *et al*. reported that OAB did not affect sexual dysfunction among sexually active young women^19^. In addition, Zachariou *et al*. reported that OAB treatment with tolterodine was associated with improved sexual function^20^. Thus, we were interested in whether OAB severity is associated with sexual dysfunction and whether OAB treatment can improve sexual dysfunction.

Thus, the primary objective of this study was to compare vacularization and blood flow perfusion of the bladder wall between the OAB and the asymptomatic controls. In addition, we were interested in elucidating (1) whether our novel method of estimating BWV is a reliable and good method for assessing female OAB and (2) the impact of tolterodine on vacularization and blood flow of the bladder, BWT, BWV and female sexual function.

## Materials

Between September 2010 and March 2016, all women with OAB were invited to participate in this prospective study. Only the data for women who completed 12 weeks of tolterodine ER (4 mg per day) treatment were included in the analysis. All women underwent transvaginal three-dimensional power Doppler ultrasonographic examinations to measure three sites (trigone, anterior wall and dome of the bladder) of the BWT, BWV, vascularization index (VI), flow index (FI) and vascularization-flow index (VFI) of the bladder wall; they also answered Patient Perception of Bladder Condition, Urgency Severity Scale, Overactive Bladder Symptoms Scores (OABSS), Urogenital Distress Inventory-6, Incontinence Impact Questionnaire-7 and Female Sexual Function Index (FSFI) questionnaires^21^ before and after 12 weeks of treatment. The study was approved by the Research Ethics Review Committee of Far Eastern Memorial Hospital. Informed consent was obtained from all subjects.

The inclusion criteria were as follows: (1) female patients who were at least 18 years old with at least one month history of OAB symptoms, including urinary urgency with or without urgency incontinence and (2) an average of > 8 micturitions in 24 hours. The exclusion criteria included clinically significant dysuria, severe stress urinary incontinence or mixed urinary incontinence with dominant stress incontinence, regular urethral catheterization or intermittent self-catheterization, urinary retention, urinary tract infection in the previous two weeks, bladder calculus, a history of pelvic radiotherapy or past/existing malignant pelvic tumors, uncontrolled narrow-angle glaucoma, intestinal obstruction and other symptoms that are contraindications for antimuscarinic medication^22^.

All ultrasonographic data were acquired using a Voluson 730 (GE Medical Systems, Zipf, Austria) ultrasound machine equipped with a 5-MHz transvaginal transducer. Similar preset power Doppler ultrasound settings were used for all examinations: pulse repetition frequency, 0.9 kHz; gain, 0.8; wall motion filter, low 1; and quality, normal.

In ultrasonography, the outer hyperechogenic layer of the bladder wall represent the adventitia, and the inner hyperechogenic layer of the bladder wall represent the mucosa/submucosal tissue^8^. The detrusor muscle appears hypoechogenic and is located between the hyperechogenic lines of the adventitia and mucosa. Measurement of all three layers represents BWT^8^, and measurement of the detrusor muscle only represents detrusor wall thickness^8^. Because the fluid instilled into the bladder would cause a decrease of the BWT when the bladder volume exceeded 50 mL^23^. Measurements of BWT were performed after emptying the bladder and perpendicular to the bladder mucosa, from the outer layer of the adventitia to the inner layer of the mucosa^8^. The post-void residual urine volume was checked to ensure that it was less than 50 mL^24^. If the post-void residual urine volume was more than 50 mL, repeated emptying was requested to ensure that it was less than 50 mL, if possible. The BWT was measured three times at each location, and the mean value was used for statistical analysis.

**Figure 1:**
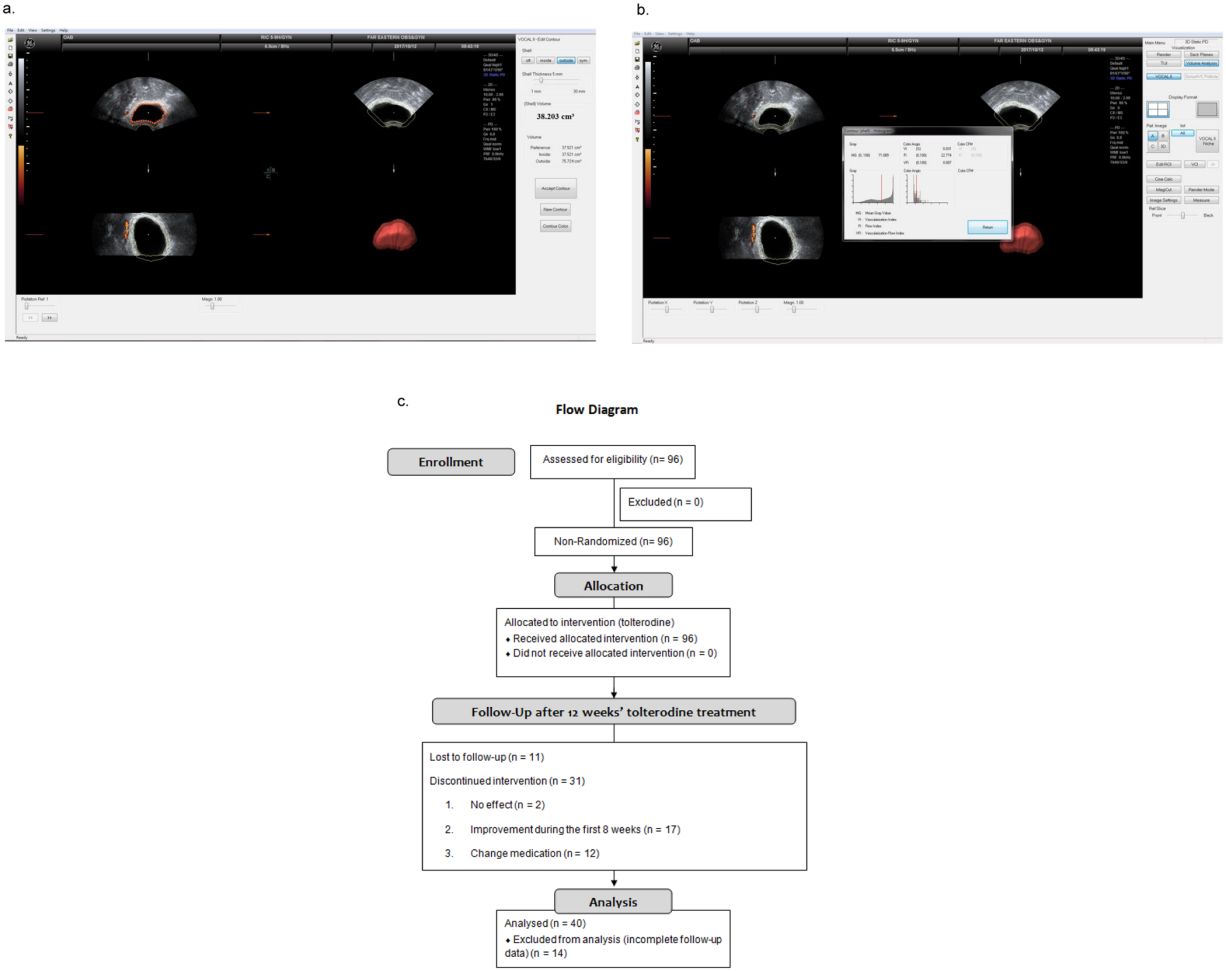
(**a**) Bladder wall volume. (**b**) Bladder wall power Doppler flow indices. (**c**) Flow chart of consecutive women with overactive bladder syndrome who underwent tolterodine treatment.

For three-dimensional ultrasound, we scanned transvaginally. Once a satisfactory grayscale image (longitudinal view) of the urethra and bladder had been obtained, the urethra was centralized onscreen, and a three-dimensional power Doppler dataset for the bladder and urethra was acquired, ensuring that the complete volume of the bladder area had been captured. The truncated sector defining the region of interest was adjusted, and the sweep angle was set to 85° to ensure that as complete a bladder wall scan as possible was obtained. Volumes of satisfactory quality were stored on the hard disk for later analysis. The VOCAL (Virtual Organ Computer-aided Analysis, Kretztechnik AG) imaging program was used to calculate BWV (i.e., shell volume in Fig. 1a) and bladder wall power Doppler flow indices (Fig. 1b).

The outer bladder volume was derived from an outward extension of inner bladder volume with a rounding integer BWT (i.e., shell thickness [mm] in Fig. 1a). BWV (i.e., shell volume in Fig. 1a) was derived by subtracting the inner bladder volume from the outer bladder volume^17^. The VI, measuring the ratio of the number of color voxels to the number of all voxels, is thought to represent the blood vessels density and was expressed as a percentage (%) of the bladder wall volume^24^. The FI represents the intensity of flow at the time of the three-dimensional sweep over the bladder wall and is thought to express the average flow intensity^25^. The VFI is a combination of vascularization and blood flow information; it thus represents overall perfusion^25^.

Multichannel urodynamic equipment (Life-Tech, Houston, TX, USA) with computer analysis and Urovision (Urolab Janus System V, Houston, TX, USA) was used. All terminology conformed to the standards recommended by the International Continence Society and Urodynamic Society^1^. The data was interpreted by a single observer to avoid interobserver variability.

A voiding detrusor pressure at maximum flow rate (PdetQmax) of more than 35 cmH_2_O was considered a high PdetQmax, while 10–35 cmH_2_O was considered a normal PdetQmax, and 10 cmH_2_O or less was considered a low PdetQmax^26^. In this study, patients with the volume at strong desire to void >300 mL, a normal PdetQmax or a low PdetQmax but with a maximum flow rate (Qmax) >12 mL/s, and a post-void residual less than 150 mL were considered urodynamically normal^26^. Additionally, women with a high PdetQmax (>35 cmH_2_O) and a low Qmax (<12 mL/s) were suggested to have bladder outlet obstruction in this study^26^.

The FSFI includes six domains: desire, arousal, lubrication, orgasm, satisfaction and pain^21^. Higher FSFI scores represented greater positive effects on sexual function. Women without sexual intercourse in recent one month did not be requested to complete the FSFI questionnaires^21^. Besides, we used the criteria of FSFI total score of> 26.55 as a diagnosis of sexual dysfunction^27^.

All methods in this study were carried out in accordance with relevant guidelines and regulation. STATA software (Version 11.0; Stata Corp, College Station, TX, USA) was used for statistical analyses. The chi-square test and Wilcoxon rank-sum test were used as appropriate. A *p* value of less than 0.05 was considered statistically significant.

Since one of the objectives of this study was to estimate the difference of bladder wall VI between the asymptomatic controls and the OAB women, a priori study for the first 15 women in each group showed a mean VI of 0.17 ± 0.20 and 0.64 ± 0.79, respectively. To detect a difference in the VI based on the information above, we conducted a test with a significance level of 0.05 and a power of 0.9 and anticipated that groups of equal size would be required. Thus, we concluded that at least 32 subjects in each group were required to test the above hypothesis.

## Results

A total of 40 women completed 12 weeks of tolterodine treatment (Fig. 1c). The above 40 women with OAB and another 34 women without OAB were analyzed in this study. Baseline characteristics are tabulated in Table 1.

**Table 1:** Baseline data of women with overactive bladder syndrome (n = 40) and the control group (n = 34).

Variables	OAB	Control	^†^P	^‡^Coefficient of OAB	95% CI	^‡^P
	(n = 40)	(n = 34)				
Age (years)	52.5 ± 10.8	55.3 ± 8.7	0.09	—	—	—
Parity	2.4 ± 1.3	2.4 ± 1.1	0.73	0.2	−0.4 to 0.7	0.53
Body mass index (kg/m^2^)	24.7 ± 3.6	23.2 ± 5.5	0.45	1.7	−0.7 to 4.1	0.15
Diabetes mellitus	3 (8)	2 (6)	1.00	—	—	—
PPBC	4.3 ± 1.0	1.2 ± 0.5	<0.0001	3.0	2.6 to 3.4	<0.001
OABSS	5.8 ± 3.5	1.5 ± 0.9	<0.0001	4.4	3.1 to 5.6	<0.001
USS	2.2 ± 1.0	0.8 ± 0.5	<0.0001	1.4	1.0 to 1.8	<0.001
UDI-6	5.8 ± 3.5	0.9 ± 1.0	<0.0001	4.8	3.6 to 6.1	<0.001
IIQ-7	7.0 ± 5.4	0.4 ± 1.7	<0.0001	6.2	4.4 to 8.1	<0.001
**Color Angio**						
VI (%)	0.40 ± 0.57	0.17 ± 0.22	0.003	0.24	0.03 to 0.46	0.03
FI (0, 100)	30.3 ± 6.9	26.6 ± 7.0	0.01	3.2	−0.2 to 6.5	0.06
VFI (0, 100)	0.15 ± 0.28	0.05 ± 0.08	0.003	0.10	0.001 to 0.21	0.047
**BWT**						
Trigone (cm)	0.56 ± 0.13	0.47 ± 0.11	0.004	0.08	0.03 to 0.14	0.005
Anterior wall (cm)	0.54 ± 0.12	0.49 ± 0.10	0.09	0.05	−0.00 to 0.11	0.07
Dome (cm)	0.54 ± 0.12	0.48 ± 0.11	0.07	0.05	−0.00 to 0.10	0.07
Average BWT (cm)	0.55 ± 0.10	0.48 ± 0.09	0.02	0.06	0.01 to 0.11	0.01
BWV (cm^3^)	35.0 ± 15.5	42.3 ± 23.9	0.24	−6.9	−16.7 to 2.8	0.16
**FSFI**						
Desire	4.3 ± 1.4	4.4 ± 0.9	0.88	−0.1	−1.3 to 1.0	0.82
Arousal	11.2 ± 3.3	10.2 ± 4.5	0.74	0.7	−2.6 to 4.0	0.66
Lubrication	14.3 ± 4.1	15.4 ± 3.9	0.43	−2.0	−5.4 to 1.5	0.25
Orgasm	10.0 ± 2.8	11.4 ± 2.7	0.16	−1.6	−4.1 to 0.8	0.19
Satisfaction	11.8 ± 3.0	11.7 ± 2.1	0.65	−0.4	−2.8 to 2.0	0.72
Pain	12.1 ± 4.2	11.8 ± 3.3	0.56	0.7	−2.7 to 4.1	0.67
Total FSFI	23.7 ± 4.9	24.3 ± 5.2	0.76	−1.0	−5.5 to 3.4	0.65

Values are expressed as the mean ± standard deviation or number (percentage). BWT = bladder wall thickness; BWV = bladder wall volume; FI = flow index; FSFI = Female Sexual Function Index; IIQ-7 = Incontinence Impact Questionnaire-7; OABSS = Overactive Bladder Symptoms Scores; PPBC = Patient Perception of Bladder Condition; UDI-6 = Urogenital Distress Inventory-6 Questionnaire; USS = Urgency Severity Scale; VFI = vascularization flow index; VI = vascularization index.

^†^By Wilcoxon rank-sum test.

^‡^Multivariable linear regression analysis adjusted by age (OAB = 1, control = 0).

From the above 74 women (i.e., 40 women with OAB and another 34 women without OAB), the intraclass correlations for VI, FI and VFI were 0.663 (95% confidence interval [CI] = 0.556 to 0.771), 0.625 (95% CI = 0.510 to 0.741) and 0.641 (95% CI = 0.529 to 0.753), respectively, all p < 0.0001.

The intraclass correlations for trigone BWT, dome BWT, anterior BWT, average BWT and BWV were 0.680 (95% CI = 0.577 to 0.783), 0.613 (95% CI = 0.496 to 0.731), 0.639 (95% CI = 0.526 to 0.751), 0.775 (95% CI = 0.697 to 0.853) and 0.878 (95% CI = 0.832 to 0.924), respectively, all p < 0.0001.

**Figure 2:**
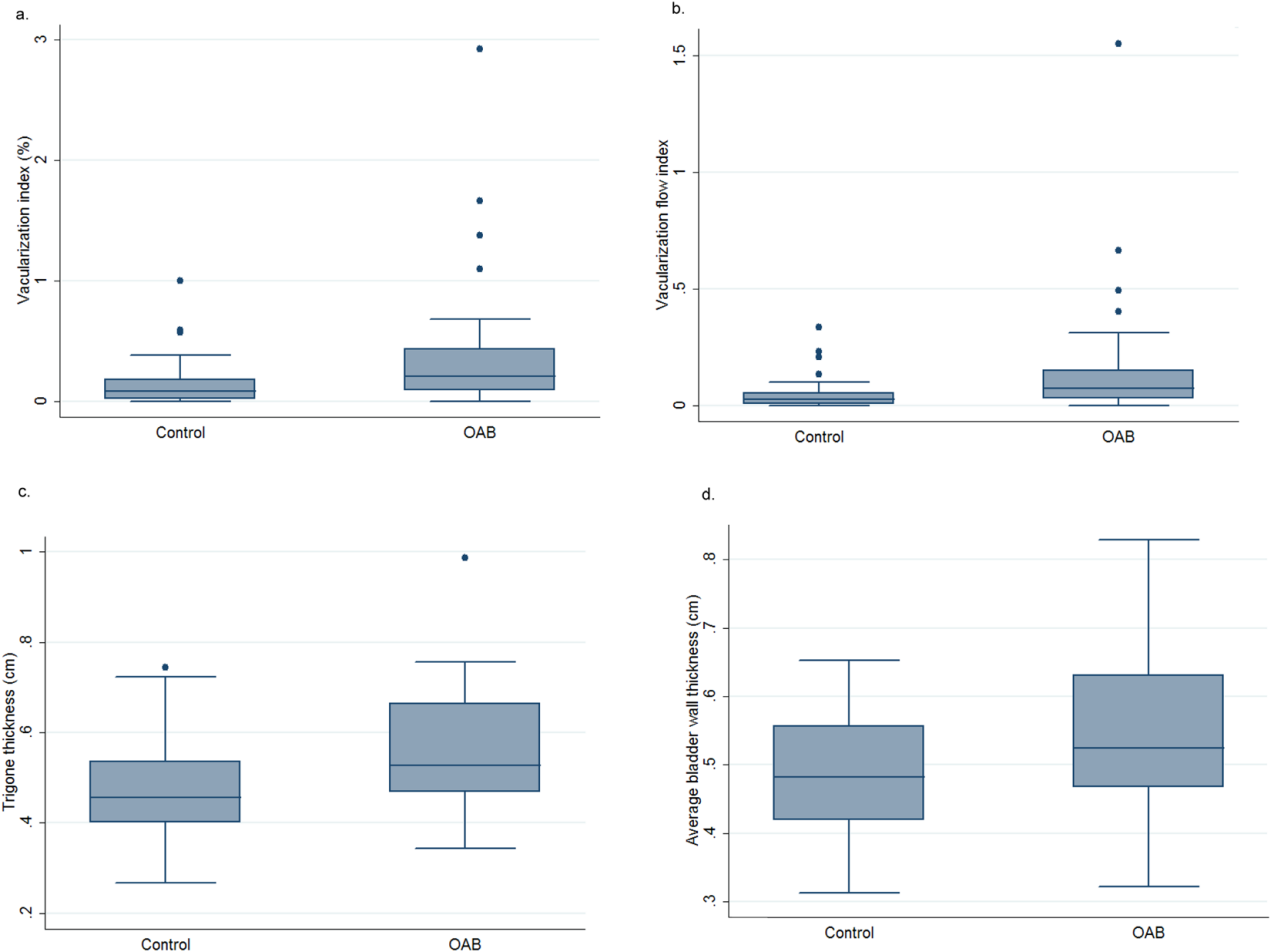
The box plots of (**a**) vasularization index (VI), (**b**) vascularization flow index (VFI), (**c**) trigone bladder wall thickness and (**d**) average bladder wall thickness between the women with overactive bladder and the control group.

Comparisons of clinical data were tabulated in Table 1. Women with OAB had higher VI (Fig. 2a) and VFI (Fig. 2b), and thicker trigone (Fig. 2c) and average BWT (Fig. 2d, all p < 0.05, Table 1). Even after multivariable linear regression with age adjustment, the above findings remained statistically significance (Table 1).

**Figure 3:**
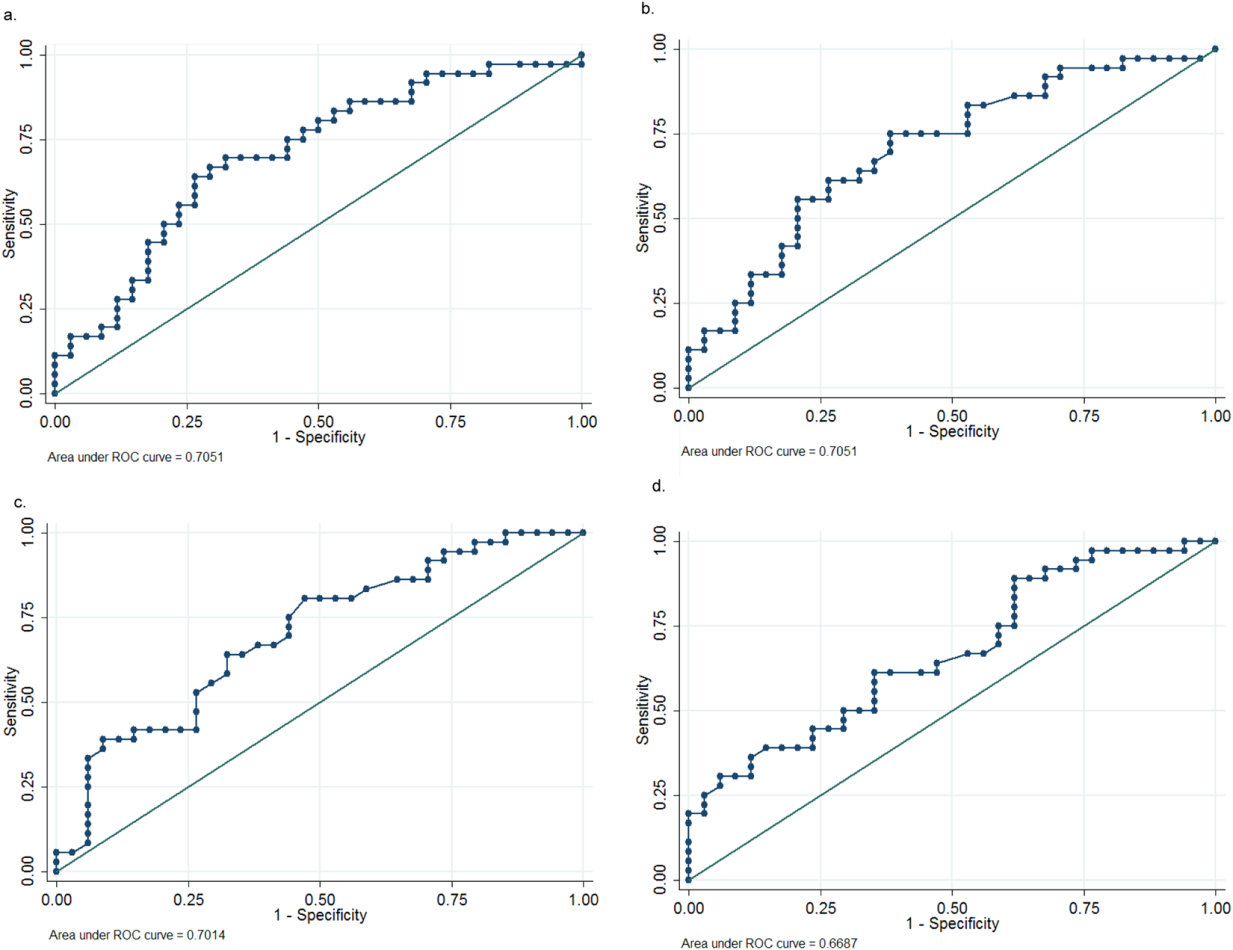
The receiver operating characteristic (ROC) curves of using (**a**) vasularization index (VI), (**b**) vascularization flow index (VFI), (**c**) trigone bladder wall thickness and (**d**) average bladder wall thickness to predict overactive bladder syndrome.

The following optimum cut-off values to predict OAB were determined using ROC analysis: (1) VI (%) ≥ 0.16, which has an area under the receiver operating characteristic (ROC) curve of 0.71 (95% CI = 0.58 to 0.83; sensitivity = 63.9%, specificity = 73.5%, Fig. 3a); (2) VFI ≥ 0.032, which has an area under the ROC curve of 0.71 (95% CI = 0.58 to 0.83; sensitivity = 75.0%, specificity = 61.8%, Fig. 3b); (3) trigone BWT ≥ 0.47 cm, which has an area under the ROC curve of 0.70 (95% CI = 0.58 to 0.82; sensitivity = 80.6%, specificity = 52.9%, Fig. 3c); and (4) average BWT ≥ 0.45 cm, which has an area under the ROC curve of 0.67 (95% CI = 0.54 to 0.80; sensitivity = 88.9%, specificity = 38.2%, Fig. 3d).

Correlation analysis showed that the strongest correlation with VI, FI, VFI, trigone BWT or average BWT was urgency (i.e., the score of the third question of OABSS, Spearman’s rho = 0.31 to 0.39, p < 0.01, Table 2). Nonetheless, BWV did not correlated with urgency (rho = 0.05, p = 0.69, Table 2). Besides, trigone and average BWTs did not correlate with VI, FI or VFI (Table 2).

**Table 2:** Correlations between the bladder wall blood perfusion/thickness/volume with the baseline subscores of OABSS questionnaire in women with and without overactive bladder syndrome (n = 74).

Variables	VI		FI		VFI		Trigone BWT		Average BWT		BWV	
	Rho	^†^P	Rho	^†^P	Rho	^†^P	Rho	^†^P	Rho	^†^P	Rho	^†^P
OABSSQ1 (Daytime frequency)	0.05	0.68	−0.09	0.47	0.04	0.77	0.11	0.36	−0.02	0.85	0.08	0.54
OABSSQ2 (Nocturia)	0.24	0.05	0.12	0.33	0.24	0.049	0.29	0.01	0.11	0.37	−0.15	0.22
OABSSQ3 (Urgency)	0.34	0.004	0.31	0.009	0.35	0.003	0.39	0.001	0.37	0.002	0.05	0.69
OABSSQ4 (Urgency incontinence)	0.14	0.24	0.13	0.27	0.13	0.27	0.08	0.50	0.08	0.54	−0.03	0.84
Trigone BWT	−0.06	0.63	0.13	0.27	−0.03	0.80	—	—	—	—	—	—
Average BWT	−0.02	0.83	0.19	0.11	0.11	0.92	0.82	<0.0001	—	—	—	—
BWV	−0.27	0.02	−0.21	0.09	−0.26	0.03	0.39	0.0008	0.45	0.0001	—	—

Values were expressed as Spearman’s rank correlation coefficients. Abbreviations were the same as Table 1. ^†^By Spearman’s rank correlation.

Correlation analyses between the bladder wall blood perfusion/thickness with the baseline urodynamic parameters in women of OAB were shown in Table 3. Pressure transmission ratio was found to be negatively correlated with VI (rho = −0.35) and VFI (rho = −0.34), and positively correlated with average BWT (rho = 0.44) and BWV (rho = 0.46, Table 3). The other correlation analyses did not show any statistical significance.

**Table 3:** Correlations between the bladder wall blood perfusion/thickness with the baseline urodynamic parameters in women of overactive bladder syndrome (n = 40).

Variables	Baseline	VI		FI		VFI		Trigone BWT		Average BWT		BWV	
		Rho	^†^P	Rho	^†^P	Rho	^†^P	Rho	†P	Rho	^†^P	Rho	^†^P
USI	27 (68)	−0.16	0.36	−0.01	0.97	−0.14	0.42	−0.06	0.71	−0.01	0.96	0.15	0.40
BO	33 (83)	0.02	0.90	−0.02	0.93	0.05	0.80	0.16	0.37	0.14	0.41	0.16	0.37
DO	12 (30)	0.07	0.70	0.10	0.58	0.10	0.58	0.11	0.54	0.19	0.27	0.18	0.31
BOO	8 (20)	−0.11	0.53	0.01	0.94	−0.10	0.56	0.22	0.21	0.06	0.71	0.05	0.76
Normal urodynamic finding	2 (5)	0.09	0.63	−0.04	0.83	0.06	0.73	−0.07	0.70	−0.17	0.33	−0.24	0.16
Pad weight (g)	23.6 ± 47.6	−0.13	0.45	0.10	0.56	−0.09	0.60	0.18	0.31	0.13	0.48	0.20	0.26
Qmax (mL/s)	18.3 ± 9.6	−0.02	0.93	0.06	0.72	−0.04	0.81	−0.01	0.97	0.11	0.53	0.02	0.92
VV (mL)	211 ± 100	−0.07	0.69	−0.00	0.98	−0.09	0.59	0.07	0.69	−0.03	0.88	0.07	0.68
PVR (mL)	119 ± 106	−0.18	0.30	−0.13	0.45	−0.18	0.30	0.04	0.81	0.02	0.91	0.32	0.06
SD (mL)	226 ± 84	−0.01	0.94	−0.12	0.48	−0.04	0.80	−0.15	0.38	0.01	0.95	0.15	0.40
PdetQmax (cmH_2_O)	33.7 ± 22.4	−0.33	0.07	−0.18	0.34	−0.35	0.054	0.03	0.88	−0.03	0.87	0.11	0.55
MUCP (cmH_2_O)	74.7 ± 37.6	−0.32	0.07	−0.11	0.52	−0.33	0.053	−0.06	0.75	0.12	0.50	−0.11	0.99
FPL (cm)	2.9 ± 0.8	0.06	0.73	0.31	0.08	0.10	0.57	0.11	0.53	0.11	0.55	−0.01	0.97
PTR (%)	100.2 ± 46.3	−0.35	0.04	−0.15	0.39	−0.34	0.049	0.21	0.23	0.44	0.01	0.46	0.007

Values were expressed as mean ± standard deviation, number (percentage) or Spearman’s rank correlation coefficients. BO = bladder oversensitivity; BOO = bladder outlet obstruction; DO = detrusor overactivity; FPL = functional profile length; MUCP = maximum urethral closure pressure; PdetQmax = detrusor pressure at maximum flow rate; PTR = pressure transmission ratio at maximum urethral pressure; PVR = postvoid residual; Qmax = maximum flow rate; SD = volume at strong desire to void; USI = urodynamic stress incontinence; VV = voided volume. The other abbreviations were the same as Table 1. ^†^By Spearman’s rank correlation.

After 12 weeks of treatment, the scores on the Patient Perception of Bladder Condition, OABSS, Urogenital Distress Inventory-6 and Incontinence Impact Questionnaire-7 questionnaires were improved; however, the score of FSFI questionnaire, VI, FI, and VFI, all sites of BWT and BWV did not differ before and after treatment (Table 4). Similarly, among the subgroup of OAB women with detrusor overactivity (n = 12, Table 4), the scores on the Patient Perception of Bladder Condition, OABSS and Urogenital Distress Inventory-6 questionnaires were improved; however, the score of FSFI questionnaire, VI, FI, and VFI, all sites of BWT and BWV did not differ before and after treatment (Table 4).

**Table 4:** Comparison of baseline data and changes from baseline after 12 week of tolterodine treatment for women with overactive bladder syndrome (n = 40).

Variables	Women with OAB (n = 40)			All women with OAB and DO (n = 12)		
	Baseline	Posttreatment	^†^*p*	Baseline	Posttreatment	^†^*p*
PPBC	4.3 ± 1.0	2.6 ± 1.2	<0.0001	4.6 ± 1.0	3.0 ± 1.2	0.02
OABSS	5.8 ± 3.5	4.3 ± 3.0	0.003	7.5 ± 4.4	5.1 ± 3.8	0.02
USS	2.2 ± 1.0	1.4 ± 0.9	0.13	2.6 ± 1.3	2.1 ± 0.9	0.16
UDI-6	5.8 ± 3.5	2.9 ± 2.6	0.0003	6.2 ± 3.5	3.1 ± 3.2	0.04
IIQ-7	7.0 ± 5.4	3.4 ± 4.2	0.0006	6.4 ± 5.1	3.6 ± 4.9	0.14
**Color Angio**						
VI (%)	0.40 ± 0.57	0.39 ± 0.91	0.84	0.39 ± 0.45	0.26 ± 0.31	0.72
FI (0, 100)	30.3 ± 6.9	28.8 ± 9.6	0.54	29.9 ± 2.8	30.1 ± 9.3	0.93
VFI (0, 100)	0.15 ± 0.28	0.17 ± 0.52	0.64	0.13 ± 0.18	0.10 ± 0.14	0.66
**BWT**						
Trigone (cm)	0.56 ± 0.13	0.57 ± 0.13	0.54	0.56 ± 0.13	0.61 ± 0.15	0.13
Anterior wall (cm)	0.54 ± 0.12	0.52 ± 0.11	0.58	0.57 ± 0.09	0.54 ± 0.10	0.42
Dome (cm)	0.54 ± 0.12	0.53 ± 0.11	0.97	0.58 ± 0.08	0.54 ± 0.11	0.15
Average BWT (cm)	0.55 ± 0.10	0.54 ± 0.10	0.83	0.57 ± 0.09	0.56 ± 0.10	0.93
BWV (cm^3^)	35.0 ± 15.5	43.8 ± 24.0	0.25	37.8 ± 13.7	46.0 ± 26.7	0.42
**FSFI**						
Desire	4.3 ± 1.4	4.7 ± 1.0	0.55	5.7 ± 0.6	5.3 ± 0.6	0.32
Arousal	11.2 ± 3.3	10.8 ± 3.9	0.54	12.3 ± 2.1	12.0 ± 2.0	0.78
Lubrication	14.3 ± 4.1	13.4 ± 4.7	0.47	15.0 ± 1.7	14.7 ± 3.8	0.79
Orgasm	10.0 ± 2.8	9.9 ± 3.3	0.53	11.3 ± 1.2	10.7 ± 2.1	0.78
Satisfaction	11.8 ± 3.0	11.9 ± 3.3	0.14	13.3 ± 1.5	13.0 ± 1.7	0.32
Pain	12.1 ± 4.2	12.2 ± 4.0	0.88	15.0 ± 0.0	12.7 ± 3.2	0.16
Tot FSFI	23.7 ± 4.9	23.7 ± 5.8	0.61	25.7 ± 4.4	27.5 ± 2.4	0.59

Values are expressed as the mean ± standard deviation. The abbreviations are the same as in Tables 1 and 3. ^†^By Wilcoxon sign-rank test.

Twenty women in the OAB group and 9 women in the control group had baseline FSFI data. Using the criteria of FSFI total score of > 26.55 as a diagnosis of sexual dysfunction^26^, 7 of 20 (35%) women in the OAB group and 3 of 9 (33%) women in the control group were found to have sexual dysfunction (p = 1.00). After tolterodine treatment, two women became having no sexual dysfunction, two women became having sexual dysfunction, and 5 women had persistent sexual dysfunction (McNemar’s test, p = 1.00).

## Discussion

In this study, we found that women with OAB had higher VI (Fig. 2a) and VFI (Fig. 2b), compared with the asymptomatic control. In addition, VI and VFI were positively correlated with urgency (Spearman’s rho = 0.34 and 0.35, p = 0.004 and 0.003, respectively, Table 2). Urgency is the core symptoms of female OAB^28^. That is, woman with OAB has a higher vascularization and overall blood perfusion of the bladder than woman without OAB. It was reported that C reactive protein was elevated in women with OAB-wet^29^, and hinted that inflammation might play a role in the etiology of OAB. Besides, stimulated macrophages and keratinocytes can produce high levels of proangiogenic factors in the inflammatory response; thus, the level of angiogenesis often correlates with inflammation^30^. The above might partly explain the positive correlation between OAB and higher vascularization of the bladder in our study. However, contrarily to our study, chronic bladder ischemia was reported to be associated with OAB^2,3,5–7^.

Kojima *et al*. used ultrasound-estimated bladder weight as a measure of bladder hypertrophy^17^. In this study, the novel method to estimate BWV had a good intraclass correlation of 0.878 (95% CI = 0.832 to 0.924). In addition, the intraclass correlation of BWV is better than that of BWT (intraclass correlation = 0.613 to 0.775). Thus, the finding might mean that our novel method to estimate BWV is a good method, and could be used in the studies of bladder wall thickness or bladder wall weight.

In this study, we found that trigone BWT and average BWT were positively correlated with baseline urgency (Spearman’s rho = 0.39 and 0.37, p = 0.001 and 0.002, respectively, Table 2), but not anterior wall BWT, dome BWT and BWV. It has been reported that BWT is associated with OAB^9,11^, detrusor overactivity^8,13,15^, detrusor pressure and lower cystometric capacity^13^. In our study, our data also demonstrated that trigone and average BWTs is positively correlated with urgency severity (Table 2). Yilmaz *et al*. also reported a good correlation between BWT and OABSS (r = 0.48, p = 0.002)^12^.

It has been reported that BWT decreases after antimuscarinic treatment^8,11,14^. Nonetheless, in our study, the changes in BWT and BWV did not differ between baseline and after treatment (Table 4). Similarly, Robinson *et al*. reported that there was no significant reduction in BWT between the solifenacin and the placebo groups^10^.

Pressure transmission ratio at maximum urethral pressure was found to be negatively correlated with VI and VFI in the OAB group (Table 3). We did not find any similar report. Low pressure transmission ratio was associated with urethral hypermobility^31^. Thus, our result might hint that OAB women with urethral hypermobility have high vasularization and overall perfusion of the bladder wall.

Tolterodine was reported to be associated with a decrease in arterial stiffness^32^. However, in our study, tolterodine failed to demonstrate any effect on bladder wall vascularization and blood perfusion owing to the lack of a difference between baseline and post-treatment in VI, FI and VFI (Table 4). Currently main medications for OAB patients included those with inhibition of detrusor contraction (such as antimuscarinics) or activation of detrusor relaxation (such as beta 3 agonist). After tolterodine treatment, significant improvements of OAB symptoms were found in our patients, but not overall perfusion and BWT (Table 4); the above discrepancy might hint that tolterodine do not treat underlying etiology of OAB. Thus, it is not a surprise that recurrence of OAB is not uncommon after discontinuation of antimuscarinics^22^.

Ultrasound for monitoring OAB treatment was reported to be not useful^5^. Rachaneni *et al*. reported that the levels of measurement error are high for a small measurement of BWT, and transvaginal ultrasound measurements have insufficient reliability and reproducibility to be an accurate diagnostic test^33^. Latthe *et al*. reported that BWT ≥ 5 mm is not a good criterion for detecting detrusor overactivity owing to its low sensitivity (43%) and specificity (62%)^15^. Rachaneni *et al*. also reported that BWT has no relationship with detrusor overactivity^16^. Together with our finding, although BWT is positively correlated with OAB severity, BWT is not a useful measure for assessing OAB severity and therapeutic efficacy.

OAB women frequently have sexual dysfunction^18^. In our study, FSFI total scores did not differ between the OAB and the control group (Table 1). Similarly, Ergenoglu *et al*. reported that OAB did not significantly affect female sexual function score^19^.

In our study, female sexual function did not improve after tolterodine treatment (Table 4). Similarly, in an Iranian study, tolterodine did not improve female sexual function^34^. However, tolterodine was reported to have a positive effect on female sexual function in some studies^20,35,36^. One study of the United States reported that tolterodine ER was associated with an improvement of the Pelvic Organ Prolapse/Urinary Incontinence Sexual Questionnaire total score, compared with placebo^35^. Similarly, one Iranian study reported that Arizona Sexual Experience Scale total score was improved after 3 months’ tolterodine treatment^36^. Recently, a Greek study reported that FSFI total scores were improved after 3 months’ tolterodine ER treatment^20^; however, a lower mean age (43 ± 8.4 years vs. 52.5 ± 10.8 years) and a lower baseline FSFI total score (17.4 ± 1.2 vs. 23.7 ± 4.9) were noted in the Greek study^20^, compared with our current study.

Prevalence of sexual dysfunction varies with race^37–40^. For example, Chinese middle-aged women reported more pain during intercourse and less sexual desire than the white middle-aged women^39^. Besides, Asian middle-aged and older women tended to report less frequent sexual activity^40^. Thus, the discrepancy about the impact of tolterodione on female sexual function between our and the above studies might be at least partly related to age and racial differences.

Limitations of this study include the limited sample size, a high drop-out rate and the lack of long-term data.

## Conclusions

Women with OAB have higher vascularization and overall perfusion of the bladder wall, and thicker bladder wall thickness, compared women without OAB. However, vascularization and overall perfusion of the bladder wall and bladder wall thickness seems not change after 12 weeks’ tolterodine treatment. Further studies might be performed to elucidate the long-term effect of antimuscarinics.

## Data Availability

The datasets generated during and/or analysed during the current study are available from the corresponding author on reasonable request.
